# Solution Self-Assembly of Coil-Crystalline Diblock Copolypeptoids Bearing Alkyl Side Chains

**DOI:** 10.3390/polym13183131

**Published:** 2021-09-16

**Authors:** Naisheng Jiang, Donghui Zhang

**Affiliations:** 1 School of Materials Science and Engineering, University of Science and Technology Beijing, Beijing 100083, China; 2 Macromolecular Studies Group, Department of Chemistry, Louisiana State University, Baton Rouge, LA 70803, USA

**Keywords:** polypeptoids, diblock copolymers, crystallization, solution self-assembly

## Abstract

Polypeptoids, a class of synthetic peptidomimetic polymers, have attracted increasing attention due to their potential for biotechnological applications, such as drug/gene delivery, sensing and molecular recognition. Recent investigations on the solution self-assembly of amphiphilic block copolypeptoids highlighted their capability to form a variety of nanostructures with tailorable morphologies and functionalities. Here, we review our recent findings on the solutions self-assembly of coil-crystalline diblock copolypeptoids bearing alkyl side chains. We highlight the solution self-assembly pathways of these polypeptoid block copolymers and show how molecular packing and crystallization of these building blocks affect the self-assembly behavior, resulting in one-dimensional (1D), two-dimensional (2D) and multidimensional hierarchical polymeric nanostructures in solution.

## 1. Introduction

Self-assembly of amphiphilic block copolymers (BCPs) in solution is one of the most fascinating phenomena in polymer physics due to the unique properties and numerous potential applications of the resulting nanostructures. The creation of various well-defined polymeric nanostructures with tailorable size and functionalities via solution self-assembly is not only useful in drug delivery, catalysis, optoelectronics and structured nanomaterials, but also provided unique perspective to understand the structural complexity and assembly rules of biomacromolecules observed in biological systems. To minimize the total free energy of the system, polymeric amphiphiles tend to self-assemble into well-defined morphologies in a selective solvent whenever the polymer concentration is above the critical micelle concentration (CMC). For coil–coil diblock copolymers in selective solvent, the most common self-assembled morphologies include spherical micelles, wormlike micelles and vesicles with a core-shell type of architecture. The thermodynamic equilibrium morphology of these self-assembled structures is described by the so-called dimensionless packing parameter, *p*, which is defined by *p* = *v*/*a*_0_*l*_c_, where *v* and *l*_c_ are the volume and the length of the solvophobic block, respectively, and *a*_0_ is the optimal surface area of the solvophilic block at the core-corona interface [1]. In many cases, the thermodynamic equilibrium with lowest free energy is not readily achieved, as the molecular exchange amongst polymeric aggregates is sluggish relative to their self-assembly process [2]. This in turn opens up opportunities to utilize different self-assembly pathways to attain uncommon solution morphologies that are kinetically trapped.

The early scaling work done by Vilgis and Halperin [3] suggested that by introducing a crystallizable block, which adds an extra driving force into the system, the self-assembly behavior of amphiphilic BCPs in solution can be significantly altered. The crystalline core confined within polymeric micelles may provide novel control options when used as seeds for further crystallization. During the past two decades, solution self-assembly of BCPs with a crystallizable block has been used as an effective method for the generation of various non-spherical polymeric micelles or nanostructures, including one-dimensional (1D) nanofibers or nanorods [4,5,6], two-dimensional (2D) nanosheets or platelets [7,8,9] or even more sophisticated hierarchical nanostructures under specific conditions [10]. This is also known as the so-called crystallization-driven self-assembly (CDSA) process where the aggregate morphology and self-assembly pathways are dominated by the epitaxial crystalline growth of macromolecular building blocks in solution. More importantly, upon crystallization, the molecular exchange is often restricted due to high free energy penalty, resulting in kinetically trapped molecular assemblies in an out-of-equilibrium state with a very long lifetime [2,11,12,13,14,15]. As a result, one can access novel polymeric nanostructures with varying morphology by controlling the self-assembly pathways during sample preparation. It has been shown that CDSA pathways and final solution morphologies of BCPs can be influenced by many factors, such as chemical composition, block ratios, polymer concentration, polymer–solvent interactions, molecular packing of the crystallizable block, annealing condition and other external stimuli [8,16,17,18,19,20,21]. In some cases, the solution self-assembly of crystallizable BCPs can proceed in a living fashion by gradually adding molecularly dissolved unimers to the pre-existing “seed” crystals, enabling the access to near monodisperse anisotropic nanostructures or hierarchical assemblies with varying levels of structural complexity in solution [5,10,19,22,23,24,25,26,27,28,29].

Non-spherical nanomaterials formed by the solution self-assembly of biocompatible macromolecules exhibit unique properties that are desirable in many biomedical and biotechnological applications, such as drug/gene delivery [30,31] and biomineralization [32]. For example, relative to spherical nanoparticles, elongated filomicelles or nano-disks can either lead to longer blood circulation time [30] or promote cell exterior binding with reduced cell uptake [33]. To date, chemists have used many crystalline polymers as the primary core-forming building blocks to facilitate the CDSA of amphiphilic BCPs in solution, including flexible linear polymers (e.g., polyethylene [15,16], poly(ε-caprolactone) [9,17,34], poly(L-lactide)) [8,21,23] and polymers with either relatively rigid backbones or bulky side groups that exhibit either crystalline or liquid crystalline (LC)-like behaviors (e.g., poly(2-(perfluorooctyl)ethyl methacrylate [35,36], poly(γ-benzyl-L-glutamate) [37], polyferrocenylsilanes [6,10,38,39] and polythiophenes [26,28]). However, considering the potential use of polymeric self-assemblies in biomedical and biotechnological applications, there is also a need of polymers that have desirable bioactivity, cytocompatibility, biodegradability and enzymatically stability.

**Figure 1:**
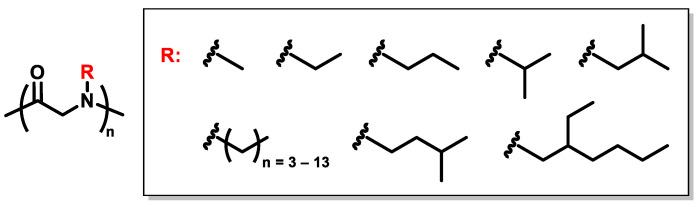
Structures of polypeptoids bearing various alkyl side chains that have been reported.

As a class of bioinspired synthetic polymers, polypeptoids featuring *N*-substituted polyglycine backbones are structural mimics of polypeptides [40,41,42,43,44,45]. Due to the absence of hydrogen bonding and stereogenic centers along the backbone (Figure 1), polypeptoids exhibit good thermal processability and solubility in various organic solvents, as well as enhanced protease stability, in sharp contrast to polypeptides. These features make polypeptoid a potential candidate for a wide variety of biomedical and biotechnological applications, such as antifouling coatings [46,47,48,49,50,51], drug/gene delivery [52,53,54,55] and biosensing [56,57,58]. Recent developments in the controlled polymerization and solid-phase synthesis have enabled access to a variety of polypeptoids with tailorable chain length, N-substituent structures, sequence, and architecture [59,60,61,62,63,64,65,66,67,68]. In the past several years, solution self-assembly of block copolypeptoids have especially attracted increasing attention due their capability to form various self-assembled nanostructures with tailorable structure, morphology, and functionality [60,67,68,69,70,71,72,73,74,75,76,77,78,79,80,81,82,83,84,85]. Given their molecular tunability, polypeptoid-based BCPs are considered as a promising biomimetic platform for macromolecular and supramolecular engineering for biomedical and biotechnological applications.

This review highlights our recent experimental findings on solution self-assembly of coil-crystalline diblock copolypeptoids bearing alkyl side chains. In the next section, we briefly discuss the synthesis and crystallization behavior of polypeptoids bearing alkyl side chains. In Section 3, we focus on the CDSA of coil-crystalline diblock copolypeptoids in solution. The effects of molecular packing, block composition and side chain architecture on CDSA, as well as the self-assembly pathways are presented. Perspectives on the solution self-assembly of coil-crystalline diblock copolypeptoids bearing alkyl side chains are followed by a brief conclusion.

## 2. Polypeptoids Bearing Alkyl Side Chains: Synthetic Methods and Their Phase Behavior

### 2.1. Controlled Ring-Opening Polymerization (ROP) for Polypeptoids

Polypeptoid-based polymers are commonly synthesized via two methods: (1) Submonomer solid-phase synthesis, and (2) ring-opening polymerization (ROP) of N-substituted glycine derived N-carboxyanhydride (R-NCA) or N-thiocarboxyanhydride (R-NTA) monomers using nucleophilic initiators (e.g., primary amine). The former method involves the growth of polypeptoid chain from the C-terminus to the N-terminus by alternating attachment of bromoacetic acid and various primary amines on a NH_2_-bearing solid support [42,86]. The stepwise synthetic method allows the access to monodispersed polypeptoids with diverse structures and precise control of chain length and sequences, which is advantageous for applications where sequence-defined copolypeptoids with complex side chain functionalities are required [40,68,69,72,73,87,88,89,90,91,92]. However, long chain polypeptoids with degree of polymerization (DP_n_) greater than 50 are difficult to obtain by the sub-monomer method. By contrast, high molecular weight polypeptoids can be synthesized by controlled ROP of R-NCA or R-NTA monomers using nucleophilic initiators such as primary amines [41,62,66,93]. A wide variety of R-NCA or R-NTA monomers have been reported for primary amine-initiated ROP of polypeptoids bearing various N-substituents in a one-pot fashion [44,62,65,66,93,94,95,96]. As this review is focused on the solution self-assembly of diblock copolypeptoids bearing alkyl side chains with relatively high molecular weight, we will mainly discuss the polymer synthesis using the controlled ROP method. The readers are referred to the previous reviews [41,42,43,44,45] for a more comprehensive view regarding the synthesis of peptoids and polypeptoids.

**Scheme 1:**
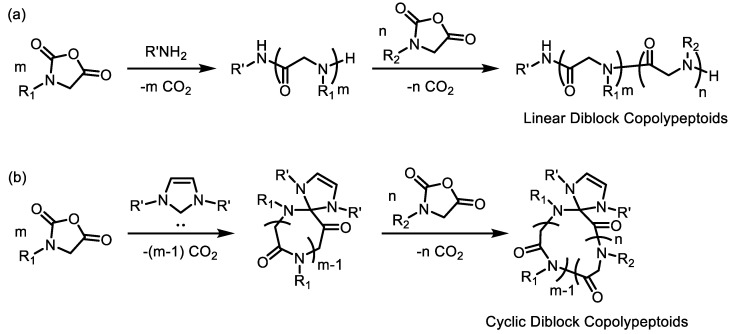
(**a**) Synthesis of linear diblock copolypeptoids via primary amine-initiated ROP of R-NCAs in a sequential manner. R’NH_2_: Primary amine. (**b**) Synthesis of cyclic diblock copolypeptoids via NHC-mediated ZROP of R-NCAs in a sequential manner.

As shown in Scheme 1, diblock copolypeptoids can be synthesized by controlled ROP of R-NCA monomers in a sequential manner using nucleophilic initiators such as primary amines. This feature has been attributed to the controlled/living nature of ROP of R-NCAs [59,61,62]. By reaching a complete conversion of the first R-NCA, the second R-NCA can be directly added to the reaction mixture as long as the product maintains a good solubility, where the entire polymerization reaction can be easily monitored by infrared (IR) spectroscopy. The actual molecular weight and block ratio of the final product can be determined by end-group analysis using ^1^H NMR spectroscopy in conjunction with matrix-assisted laser desorption/ionization time-of-flight mass spectrometry (MALDI-TOF MS), size-exclusion chromatography (SEC) or viscosity measurements. As R-NCAs are very sensitive to moisture, the use of anhydrous solvents is necessary, and the reactions are normally conducted under anhydrous condition with water content less than 30 ppm to avoid side reactions. Luxenhofer et al. have shown that the benzyl amine-initiated ROP of R-NCA, such as N-methyl N-carboxyanhydride (Me-NCA) and N-butyl N-carboxyanhydride (Bu-NCA) proceeded in a controlled manner without chain transfer or termination events, yielding well-defined homopolypeptoids with controlled molecular weights and narrow molecular weight distributions (PDI < 1.1–1.3) [61,62]. In addition, they also have shown that well-defined block copolypeptoids, e.g., poly(N-methyl glycine)-*b*-poly(N-butyl glycine) (PNMG-*b*-PNBG), can be produced by controlled ROP with sequential monomer addition [61,62]. In our previous studies [60,70,79,84], a variety of well-defined amphiphilic diblock copolypeptoids that comprised of at least one crystallizable block with relatively low PDIs have been synthesized via benzyl amine-initiated ROPs of R-NCAs in a sequential manner, allowing us to further investigate their CDSA behaviors in solution. There are several aliphatic N-substituents can be readily used for the molecular design (Figure 1). For coil-crystalline diblock copolypeptoids, Me-NCA is the most commonly used monomer to produce the amorphous PNMG block, whereas R-NCAs bearing long *n*-alkyl groups (e.g., N-octyl N-carboxyanhydride (Oct-NCA) and N-decyl N-carboxyanhydride (De-NCA)) are often used to produce the crystallizable block. Branching of the alkyl side chains can also be introduced to tune the inter- and intramolecular interactions of polypeptoid, thus allowing their molecular packing and phase behavior to be systematically tailored.

While primary amine-initiated ROP of R-NCAs yields linear diblock copolypeptoids, recent synthetic developments in the organo-mediated zwitterionic ring-opening polymerization (ZROP) of R-NCAs have also enabled access to polypeptoids with cyclic topology [59]. In previous studies, N-heterocyclic carbenes (NHCs) have been used as initiators/organo-catalysts to initiate/mediate ZROP of R-NCAs (e.g., Me-NCA, Bu-NCA Oct-NCA and De-NCA) for a variety of well-defined cyclic polypepotids with tunable molecular weight and narrow dispersity [59,63,93,97]; 1,8-Diazabicycloundec-7-ene (DBU), a bicyclic amidine that is less sensitive to air and moisture relative to NHC, has also been demonstrated capable of mediating ZROPs of R-NCAs in a similar manner [98,99]. It has been demonstrated that the ZROP proceeded through a zwitterionic propagating intermediate where the two oppositely charged chain ends are held in proximity through electrostatic interaction. Low dielectric solvents, such as tetrahydrofuran (THF) and toluene, were normally used to avoid intramolecular transamidation relative to chain propagation and ensure a controlled ZROP reaction [44]. The quasi-living nature of the organo-mediated ZROP also enable the access to well-defined amphiphilic cyclic diblock copolypeptoids by sequential monomer addition (Scheme 1b), which allow us to exploit the effect of chain topology on solution self-assembly [60].

### 2.2. Molecular Packing and Phase Behavior of Polypeptoid Homopolymers Bearing Alkyl Side Chains

As aforementioned, the self-assembly pathway and final aggregate morphology of amphiphilic coil-crystalline diblock copolymers in solution depends strongly on the molecular packing and phase behavior of the crystallizable block. Thus, it is important to first gain a thorough understanding on the general packing motifs and crystallization behavior of the corresponding homopolymers. As one would expect, molecular packing, crystallization behaviors, thermal transitions and solubility of polypeptoids highly rely on their N-substituent structures. Here, we briefly summarize the molecular packing and phase behavior of homopolypeptoids bearing alkyl side chains.

**Figure 2:**
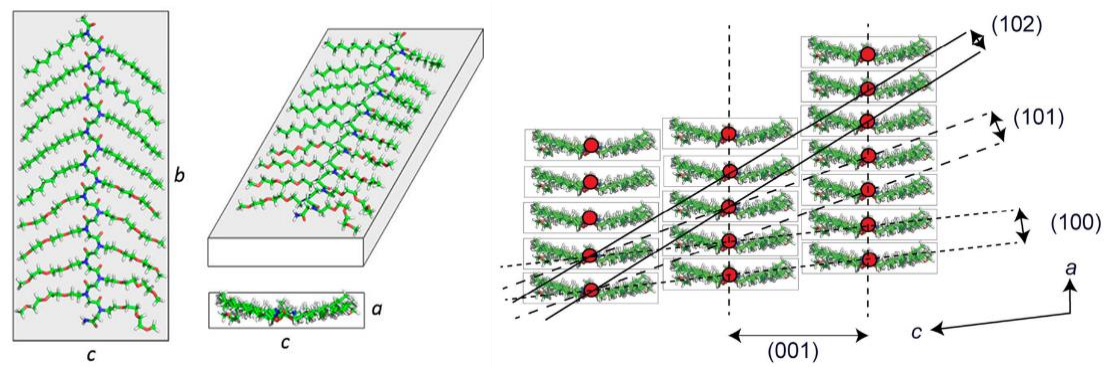
The unit cell dimensions (**left**) and supramolecular assembly of peptoid molecules with all cis-amide backbone conformation in the crystalline state (**right**). Figure reproduced from reference [108] with permission from the American Chemical Society.

Polypeptoids bearing linear aliphatic N-substituents shorter than 2 carbons are amorphous and behave as random coil-like polymers. The most famous example is poly (N-methyl glycine) (PNMG), a.k.a. polysarcosine, the simplest member of the polypeptoid family, which is amorphous and can be readily dissolved in water or alcohol [100,101,102]. As a promising biodegradable poly (ethylene glycol) (PEG)-alternative, PNMG can be served as hydrophilic component by providing steric stabilization of nanostructures, plasmonic particles or proteins for various biomedical applications, such as drug delivery and theranostics [54,102,103,104,105,106,107]. On the other hand, it has been found that polypeptoids with relatively long linear *n*-alkyl side chains (4 ≤ S ≤ 14, where S is the number of carbon atoms in the *n*-alkyl group) are highly crystalline in the solid state [89,97,108]. Using X-ray diffraction and molecular dynamics simulations, Balsara, Zuckermann and coworkers have revealed their general packing motif: Polypeptoid molecules bearing *n*-alkyl side chains tend to adopt a board-like structure in the crystalline state, where the backbone is fully extended in an all-cis backbone conformation and is approximately coplanar with the n-alkyl side chains (Figure 2) [108]. The all cis-amide backbone conformation, which is more compact and possesses a higher degree of ordering than the all-trans backbone conformation, allows for more favorable intra- and inter-molecular interactions upon crystallization/supramolecular assembly. With N backbone repeating units and S number of carbons on the *n*-alkyl side chains, the unit cell dimensions, namely *a*, *b* and *c*, follow a universal relationship, in which *a* = 0.455 nm, *b* = (0.298N + 0.035) nm and *c* = (0.186S + 0.55) nm. Note that crystallization of these board-like polypeptoids can occur at relatively low number-average degree of polymerization (e.g., DP_n_ = 9) in both solid and solution states [72,108].

**Figure 3:**
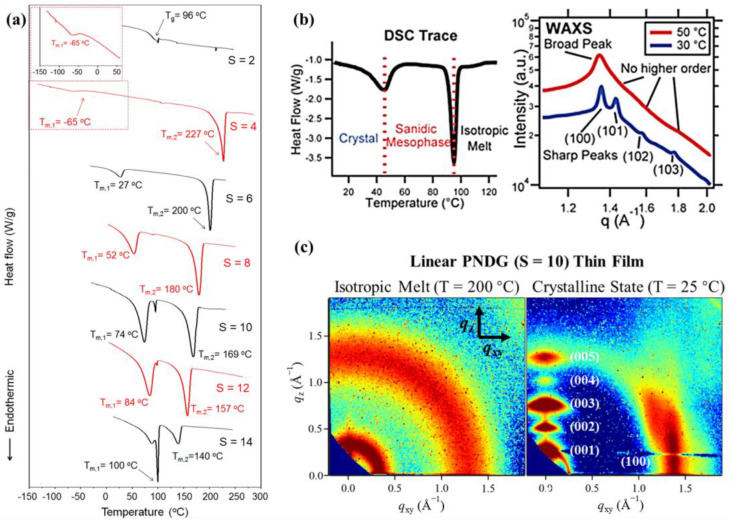
(**a**) DSC thermograms of linear polypeptoids bearing different *n*-alkyl side chains with 2 ≤ S ≤ 14 during the second heating cycle. (**b**) DSC and WAXS results of N-acetylated diblock polypeptoid bearing *n*-decyl side chains, i.e., Ac-pNdc_9_-*b*-pNte_9_. Three different phases can be identified, i.e., crystalline phase, sanidic LC mesophase and isotropic melt. (**c**) Two-dimensional GIWAXD images of 48 nm thick linear PNDG (DP_n_ = 52) thin film prepared on Si substrate measured at T = 200 °C and T = 25 °C after cooling from 200 °C, respectively. The out-of-plane (*q*_z_) and in-plane (*q*_xy_) directions are indicated by arrows. Figures reproduced from references [97,111,112] with permission from the American Chemical Society.

By using differential scanning calorimetry (DSC), Luxenhofer showed that the glass transition temperature (T_g_) of polypeptoids with 1 ≤ S ≤ 5 decreases with increasing *n*-alkyl side chain length [109], which is likely due to the plasticization effect induced by the flexible *n*-alkyl side chains [110]. The increase of *n*-alkyl side chain length also leads to an increasing tendency towards crystallization. Lee et al. found that both linear and cyclic polypeptoids bearing long linear *n*-alkyl side chains (4 ≤ S ≤ 14) exhibit two phase transitions upon temperature increase (Figure 3a) [97]. These two transition temperatures are strongly coupled and highly depend on the number of carbon atoms in the *n*-alkyl group. Using temperature-dependent X-ray scattering, Balsara, Zuckermann and coworkers further evidenced the broadening of the (100) peak and the disappearance of higher order peaks of diblock polypeptoid bearing *n*-decyl side chains during the lower-temperature transition, indicating the diminished ordering for the face-to-face stacking of the board-like polypeptoid molecules (Figure 3b) [111]. Thus, it has been suggested that the lower-temperature transition corresponds to a crystalline phase to a “sanidic” liquid crystalline (LC) mesophase transition, while the higher-temperature transition corresponds to the LC mesophase to isotropic melt transition (Figure 3b) [111]. In our very recent study on poly (N-decyl glycine) (PNDG) thin films prepared on solid substrates, we showed that both linear and cyclic PNDG exhibit two amorphous halos when heating above the isotropic melt transition temperature (Figure 3c) [112]. Interestingly, the d-spacings of these two amorphous halos are in good agreement with the theoretical molecular dimension of PNDG in an extended trans-amide backbone conformation. Therefore, it was proposed that polypeptoid molecules undergo a cis-to-trans amide backbone conformational transition when heating above the isotropic melting temperature, where the long *n*-alkyl side chains are still nearly coplanar with the polypeptoid backbone [112].

**Figure 4:**
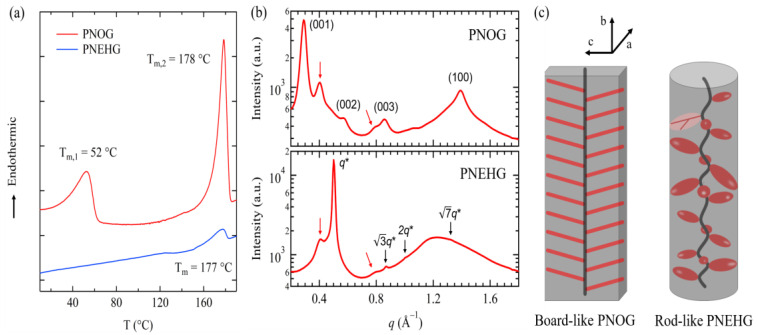
(**a**) DSC thermograms of PNOG (DP_n_ = 103) and PNEHG (DP_n_ = 100) homopolymers during the second heating cycle. (**b**) One-dimensional WAXD profiles of the PNOG and PNEHG homopolymers measured at T = 45 °C. The primary and secondary peaks associated to the Kapton windows on the sample cell are indicated by the red arrows. (**c**) Proposed molecular geometries of PNOG and PNEHG at crystalline/liquid-crystalline state. Figure reproduced from reference [84] with permission from the American Chemical Society.

Side chain engineering has long been served as an effective strategy to modulate inter and intra-molecular interactions and packing of crystallizable polymers, thus allowing their morphology, solubility, and functionality to be systematically tailored [113]. It has been found that the molecular packing and crystallization behavior of polypeptoids are turned into a different scenario when the alkyl side chains are asymmetrically branched. For example, in the case of racemic 2-ethyl-l-hexyl side chains, it was found that relatively short poly (N-2-ethyl-1-hexyl glycine) (PNEHG) molecules with DP_n_ ≤ 20 are amorphous with no first-order transition observed by DSC [90,91]. On the other hand, longer PNEHG chains, e.g., DP_n_ ≥ 100, exhibit a single first-order thermal transition with a small enthalpic change, but are still in sharp contrast with poly (N-octyl glycine) (PNOG) homopolymer which also possesses eight carbon atoms on their side chains [84,97]. Our recent WAXD measurements on PNOG and PNEHG homopolymers with similar DP_n_ (DP_n_ ≈ 100) revealed the effect of side chain branching on the molecular geometry and supramolecular assembly [84]. As shown in Figure 4b, PNOG_103_ homopolymer was found to exhibit typical reflection peaks in the WAXD profile due to the side-by-side (along crystallographic *c*-axis) and face-to-face stackings (along crystallographic *a*-axis) of the board-like molecules, consistent with those observed for polypeptoids bearing linear *n*-alkyl side chains. By contrast, PNEHG_100_ exhibits a primary diffraction peak at q* = 0.50 Å^−1^ due to the distance (1.26 nm) between adjacent PNEHG backbones that are separated by the interdigitated N-2-ethyl-1-hexyl side chains, along with multiple weak higher order peaks located at √3q*, √4q*, √7q*, respectively. A broad amorphous peak near *q* = 1.2 Å^−1^ is also discernible, which is likely to arise from the interchain distance among the 2-ethyl-1-hexyl side chains. This result indicates that the PNEHG molecules are rod-like and packed into a hexagonal mesophase upon cooling from isotropic melt. Unlike PNOG chains that preferentially adopt a board-like geometry with all *n*-octyl side chains aligned in the same plane, the greater steric hindrance of the bulky racemic 2-ethyl-1-hexyl side chains makes it energetically unfavorable for the PNEHG to adopt a planar geometry. Instead, the PNEHG molecules adopt a rod-like geometry with an extended backbone conformation, which allow the side chains to orient outwardly along the backbone, thereby minimizing steric repulsion amongst the bulky branched alkyl substituents. Therefore, asymmetric branching of the aliphatic N-substituents (e.g., 2-ethyl-l-hexyl) not only suppresses the degree of crystallization, but also changes the packing motif of polypeptoids. As we will show, such feature allows us to modulate the solution self-assembly of amphiphilic diblock copolypeptoids by side chain branching pattern.

## 3. Solution Self-Assembly of Coil-Crystalline Diblock Copolypeptoids Bearing Alkyl Side Chains

### 3.1. Sample Preparation and Characterization of Coil-Crystalline Diblock Copolypeptoid Solutions

For long chain macromolecules, crystallization is a kinetically controlled process, implying that sample preparation and processing pathways have profound impacts on the final structure and crystalline morphology. For solution self-assembly of BCPs that comprised of a crystallizable block, size, shape and morphology of the final nanostructures strongly rely on the self-assembly pathways, which can be affected by many preparation/processing factors, including initial polymer concentration, solvent quality, impurity and thermal history. The interplay between aggregation and crystallization also plays a key role in determining the final solution morphology. As crystallization is strongly temperature and concentration dependent, it can be manipulated to control the self-assembly pathway. The simplest way to trigger a CDSA process is by first dissolve the BCPs molecularly in good or nonselective solvent, then changing the temperature or solvent quality to induce crystallization.

It should be noted that the solubility of individual blocks of BCPs in a given solvent is important when planning a CDSA experiment. For polypeptoids, the polymer–solvent interaction may vary significantly depending on the number of carbon atoms in their alkyl side groups (S). For example, while both PNMG (S = 1) and poly (N-ethyl glycine) (S = 2) have good solubility in water [107], poly (N-propyl glycine) with S = 3 was found to exhibit lower critical solution temperature (LCST) in water, which can phase separate when heated above the cloud point temperature (i.e., 15–25 °C) [114]. As the S value further increases, polypeptoid molecules become increasingly more hydrophobic and exhibited diminished solubility in water. Similar trend of solubility versus side chain length was also observed for polypeptoids in methanol, which showed that PNDG with S = 10 is more solvophobic than PNMG at room temperature, as evidenced by liquid contact angle measurements [79]. It was found that a wide range of polypeptoids bearing *n*-alkyl side chains with 1 ≤ S ≤ 12 can be readily solubilized in dichoromethane and chloroform [97]. Toluene and DMF can also dissolve polypeptoids with relatively short *n*-alkyl side chains (e.g., S = 4) [59,63], whereas for crystallizable polypeptoids bearing longer *n*-alkyl side chains, THF can provide good solubility at high temperatures (e.g., 50 °C or above) [97,112]. Note that the polypeptoid solubility may also depends on molecular weight and preparation history of the polymer samples [60,97,107].

Our previous studies mainly focused on the solution self-assembly of coil-crystalline diblock copolypeptoids bearing alkyl side chains in methanol. Using scattering techniques, we found that most coil-crystalline diblock copolypeptoids, e.g., PNMG-*b*-PNOG with *f*_PNOG_ ≤ 0.73 and PNMG-*b*-PNDG with *f*_PNDG_ ≤ 0.44 (where *f* is the volume fraction of the crystalline block), can be molecularly dissolved (i.e., forming unimers) at dilute concentrations in methanol by heating at high temperatures [79,84]. Cooling the methanol solution down to room temperature induces the recrystallization of PNOG or PNDG blocks. Therefore, CDSA of diblock copolypeptoids can be triggered by first heating the solution at high temperature, subsequently cooling to desired temperature and keep the solution under isothermal condition for prolonged time. Note that multiple structural changes and phase transitions can take place during these steps, thus, the solution samples must be carefully characterized with good spatial and temporal resolutions.

Various in situ and ex situ techniques can be used to monitor the structural evolution of coil-crystalline diblock copolypeptoids during the solution self-assembly, such as static light scattering (SLS), small-/wide-angle X-ray scattering (SAXS/WAXS), small-angle neutron scattering (SANS), (cryo-)transmission electron microscopy (TEM or cryo-TEM) and atomic force microscopy (AFM). While TEM and AFM can “visually” characterize the polymeric nanostructures self-assembled in real space, reciprocal scattering techniques using X-ray or neutron sources are more powerful tools in terms of providing global averaged structural information at length scales ranging from micrometers to angstroms [115]. In addition, scattering techniques are generally non-destructive and can directly characterize the self-assembled structures in solution environments. Modern synchrotron X-ray sources with high flux and small beam divergence, in conjunction with the use of hybrid pixel array detectors that allow direct photon detection (e.g., PILATUS detectors by Dectris Ltd.), also enable fast data collection (down to milliseconds per data) for dilute samples, which makes SAXS/WAXS ideally suited for probing multiscale structural evolution of BCPs as a function of time during solution self-assembly [116,117,118,119]. It should be mentioned that polypeptoid self-assemblies induced by CDSA often possess multiple levels of structural hierarchy and heterogeneity in solution (see examples below). Therefore, extreme care should be taken when interpreting the scattering data, especially for those collected from the small-angle regime. In this regard, SAXS and SANS are often complimentarily used in conjunction with other microscopic techniques to provide a compelling characterization of the solution CDSA process over a wide range of length- and time-scales.

### 3.2. Crystallization-Driven Self-Assembly of 1D nanofibrils

**Figure 5:**
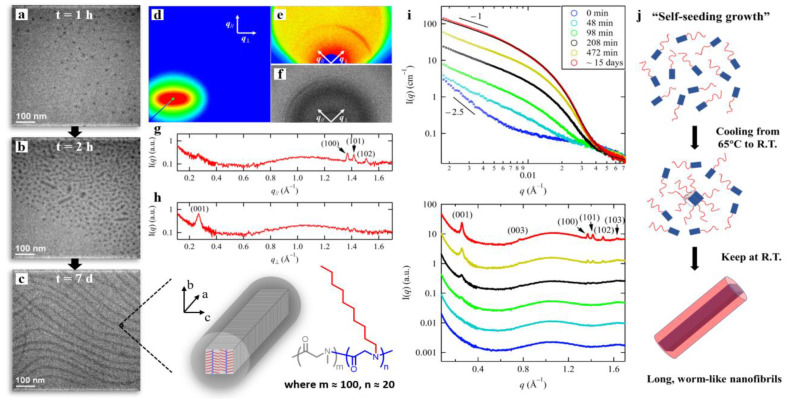
(**a**–**c**) Representative cryo-TEM images of 1 mg/mL PNMG_112_-*b*-PNDG_16_ methanol solution at different waiting time (*t*) after been cooled down to room temperature. The chemical structure of PNMG-*b*-PNDG block copolypeptoid and the schematic illustration of molecular packings of the core-forming PNDG blocks within a nanofibril are shown in the inset of (**c**), where the backbones and *n*-decyl side chains of PNDG are indicated in blue and red, respectively. (**d**–**f**) Two-dimensional SAXS (**d**), MAXS (**e**) and WAXS (**f**) images for 5 mg/mL PNMG_105_-*b*-PNDG_20_ methanol solution measured during unidirectional flow at room temperature. The directions parallel (*q*_//_) and perpendicular (*q*_⊥_) to the flow direction are indicated by arrows. The corresponding one-dimensional profiles of the MAXS/WAXS results along the *q*_//_ and *q*_⊥_ directions are plotted in (**g**) and (**h**), respectively. (**i**) SAXS (top) and MAXS/WAXS (bottom) profiles of 5 mg/mL PNMG_105_-*b*-PNDG_20_ methanol solution measured at static state at different *t*. The solid line in the SAXS profile at *t* = ~15 days corresponds to the best-fit to the data based on the cylindrical-shaped micelle model. (**j**) Schematic illustration of the proposed self-assembly mechanisms for the 1D nanofibrils (e.g., PNMG_105_-*b*-PNDG_20_) via CDSA. Figures reproduced from references [60,79] with permission from the American Chemical Society.

Long, wormlike 1D nanofibrils can be generated by the CDSA of coil-crystalline diblock copolypeptoids with relatively low volume fraction of the crystalline block in methanol. Lee et al. previously showed that PNMG_112_-*b*-PNDG_16_ comprised of a soluble PNMG block and a much shorter crystallizable PNDG slowly self-assembled into long, wormlike nanofibrils in dilute methanol with a polymer concentration of *c* = 1 mg/mL [60]. Cryo-TEM imaging revealed a morphological transition from spherical micelles to micrometer length nanofibrils during the course of seven days after the solution was cooled down to room temperature (Figure 5a–c). Similar self-assembly behavior was also found for the cyclic counterpart, i.e., cyclic-PNMG_105_-*b*-PNDG_10_, except for its relatively slower self-assembly kinetics. At higher concentrations (e.g., 10 wt%), these linear and cyclic PNMG-*b*-PNDGs form free-standing gels after the solution was cooled, which is attributed to the formation of gel network that comprised of entangled crystalline nanofibrils [70].

To better understand the crystalline packing of PNDG segment in the wormlike micelles, X-ray scattering experiments of the worm-micelle solution was conducted under unidirectional flow. Figure 5d–f shows the 2D SAXS, MAXS and WAXS data of the 5 mg/mL methanol solution containing the PNMG_105_-*b*-PNDG_20_ long, wormlike 1D nanofibrils under unidirectional flow in a capillary flow cell with a constant shear rate of ~25.6 s^−1^ near the wall. Under the influence of the flow field, the wormlike micelles were preferentially aligned parallelly with the flow direction, evidenced by the increasing anisotropy of the 2D SAXS patterns with increasing flow rate [79,120,121,122]. Meanwhile, M/WAXS analysis (Figure 5g,h) has revealed a significantly more pronounced scattering peak due to the (001) reflection in the *q*_⊥_ direction relative to that in the *q*_//_ direction, indicating that the (001) molecular packing separated by the *n*-decyl side chains (i.e., side-by-side stacking) with a *d*-spacing of *d*_001_ = 2.4 nm was aligned in the direction perpendicular to the long axis of the PNMG_105_-*b*-PNDG_20_ nanofibrils. Consistently, the scattering peaks due to (100) reflection and the associated higher order reflections from (101) and (102) planes are more notable along the *q*_//_ direction as compared to those in the *q*_⊥_ direction. According to Figure 2, the result indicates that the adjacent cis-amide backbones with a *d*-spacing of *d*_100_ = 0.46 nm due to the face-to-face stacking was aligned in a direction parallel to the long axis of the nanofibrils. These combined results support an anisotropic crystalline core structure for the long, wormlike 1D nanofibrils, as depicted in the inset of Figure 5c.

**Figure 6:**
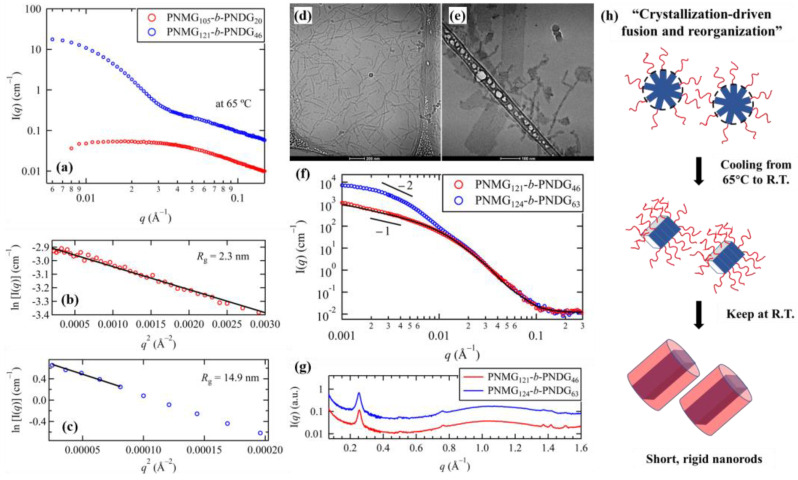
(**a**) SAXS profiles of the 5 mg/mL PNMG_105_-*b*-PNDG_20_ and PNMG_121_-*b*-PNDG_46_ methanol solutions measured at 65 °C. The corresponding Guinier plot analysis of PNMG_105_-*b*-PNDG_20_ (*R*_g_ = 2.3 nm) and PNMG_121_-*b*-PNDG_46_ (*R*_g_ = 14.9 nm) based on the criteria of *qR*_g_ < 1.3 were shown in (**b**) and (**c**), respectively. Representative cryo-TEM images for the self-assembled (**d**) PNMG_121_-*b*-PNDG_46_ and (**e**) PNMG_124_-*b*-PNDG_63_ nanostructures in methanol. (**f**) SANS intensity profile (open circles) for the self-assembled PNMG_121_-*b*-PNDG_46_ and PNMG_124_-*b*-PNDG_63_ nanostructures in deuterated methanol. The solid line in (f) corresponds to the best-fit to the data based on the cylindrical-shaped micelle model. (**g**) WAXS intensity profiles for the 5 mg/mL PNMG_121_-*b*-PNDG_46_ and PNMG_124_-*b*-PNDG_63_ in methanol. (**h**) Schematic illustration of the proposed self-assembly mechanisms for the 1D nanorods (e.g., PNMG_121_-*b*-PNDG_46_) via CDSA. Figure reproduced from reference [79] with permission from the American Chemical Society.

Time/temperature-dependent synchrotron X-ray/neutron scattering experiments were performed to further investigate the self-assembly pathway and formation mechanism of these 1D nanofibrils. High-temperature solution SAXS result shows that the PNMG_105_-*b*-PNDG_20_ polymers are well-dissolved and exist as unimers in methanol at 65 °C (Figure 6a,b), which gives a radius of gyration (*R*_g_) of 2.3 nm by Guinier plot analysis. Upon cooling the solution to room temperature, a drastic change of the scattering profile was observed (Figure 5i). At time t = 0 min, i.e., immediately after the solution was cooled down from 65°C to room temperature, the SAXS profile shows a noticeable upturn at the low *q* region, while the overall intensity is still relatively weak. Such intensity upturn with a *q*^−2.5^ dependence is attributed to the formation of polymer aggregates, i.e., the “seeds”, at the very early stage after the solution was cooled down to room temperature. With increasing of time, (001), (100) and their higher order reflections started to appear after ~100 min and intensify over time. Meanwhile, at the low *q* regime in SAXS spectra, the dependence of intensity over *q* gradually changed over from I ~ *q*^−2.5^ to *q*^−1.5^, and eventually to *q*^−1^ after ~400 min, while the overall absolute SAXS intensity continue to increase until a final state was reached. We attribute this increase in the SAXS intensity mainly due to the one-dimensional elongation of the nanofibrils, consistent with the time-dependent cryo-TEM results shown in Figure 5a–c. Note that the SAXS profile of the final PNMG_105_-*b*-PNDG_20_ nanofibrils at t = 15 days can be well-fitted by using the scattering model for core-corona cylindrical micelles developed by Pedersen and co-workers [123,124], which gives a core radius (*R*_c_) of 3.3 ± 0.2 nm and a radius of gyration of the corona chains of 3.8 ± 0.2 nm. The average diameter of the nanofibrils is then estimated to be approximately 23.4 nm, in good agreement with the cryo-TEM result.

The above results clearly show that the elongation of PNMG_m_-*b*-PNDG_n_ nanofibrils with relatively low volume fraction of PNDG segments (i.e., m ≈ 100 and *n* ≈ 20) is induced by the face-to-face stacking of PNDG backbones along the crystallographic a-axis (i.e., the (100) packing), while the cross-sectional dimension (or lateral diameter) of the nanofibrils is determined by the backbone length of PNDG and the (001) packing along the crystallographic c-axis. Based on the time-dependent SAXS/WAXS and cryo-TEM results, it is reasonable to conclude that the 1D nanofibrils formation is mainly governed by the so-called “self-seeding growth” mechanism [6,9,13,19,28,125,126], which involves the initial formation of a few small “seed” crystals followed by the preferential addition of the unimers to the crystalline front (Figure 5j). Apparently, for board-like PNDG molecules, the creation of anisotropic crystalline core requires preferential addition of the unimers to a certain crystallographic facet, instead of adding unimers equally in all directions. As we found, the face-to-face stacking of the PNDG segment is more favored over the side-by-side packing during the seeded growth process, resulting in the unidirectional elongation of long wormlike micelles.

### 3.3. Effect of Block Composition on the Solution Self-Assemblies

For amphiphilic coil-crystalline BCPs, increasing the volume fraction or DP_n_ of the solvophobic crystalline block often leads to drastic changes in the self-assembly pathway and final aggregate morphology in solution (Figure 6d,e) show the cryo-TEM images for the self-assembled PNMG_121_-*b*-PNDG_46_ (*f*_PNDG_ = 0.61) and PNMG_124_-*b*-PNDG_63_ (*f*_PNDG_ = 0.68) nanostructures in 5 mg/mL methanol solution. Unlike the long, wormlike nanofibrils formed by CDSA of PNMG-*b*-PNDGs with relatively low PNDG volume fractions (i.e., *f*_PNDG_ = 0.44), the PNMG_121_-*b*-PNDG_46_ having an intermediate PNDG volume fraction self-assembled into rigid rod-like structures with much shorter length (~100–400 nm) under identical sample preparation condition. The best-fit to the SANS data of PNMG_121_-*b*-PNDG_46_ nanorods (Figure 6f) using cylindrical-shaped polymer micelle model gives a PNDG core radius of *R*_c_ = 6.8 ± 0.2 nm, which is two times larger than that for PNMG_105_-*b*-PNDG_20_ nanofibrils. The discrete reflection peaks due to the face-to-face and side-by-side packing of PNDG were also observed by WAXS, suggesting the occurrence of highly ordered crystalline structure of core-forming PNDG blocks. Assuming the crystalline packing of the PNDG segments in the 1D nanorods are identical to that in the 1D nanofibrils, the cross section of the former core would have at least 4 PNDG molecules stacked side-by-side in a fully extended cis-amide backbone conformation.

As the volume fraction of PNDG segments is further increased, cryo-TEM revealed the predominant presence of 2D nanosheets in addition to some short nanorods for the PNMG_124_-*b*-PNDG_63_ methanol solution. The majority of the nanosheets exhibit a rectangular shape that is ~100 nm in width and several hundreds of nm in length. The average thickness of the PNMG_124_-*b*-PNDG_63_ nanosheets was estimated to be ~14 nm based on AFM analysis. WAXS analysis of the PNMG_124_-*b*-PNDG_63_ solution (Figure 6g) revealed notable diffraction peaks due to side-by-side packing of PNDG. However, the (100) reflection and the associated higher order (101) and (102) reflections are barely discernible in the WAXS region, indicating the relatively poor molecular ordering of adjacent PNDG backbones along the crystallographic a-axis. This result suggests that the formation of PNMG_124_-*b*-PNDG_63_ nanosheets is mainly driven by the side-by-side molecular packing along the crystallographic c-axis, while the face-to-face molecular packing along the a-axis is significantly diminished. We postulate that the length of the nanosheets is determined the side-by-side packing of PNDG segments along the crystallographic c-axis, whereas the width of the nanosheets is resulted from the face-to-face stacking of the PNDG segments. This picture is consistent with recent cryo-electron microscopy and molecular dynamic simulation studies on crystallizable diblock copolypeptoid nanosheets [127]. From WAXS analysis, the distance of adjacent PNDG backbones separated by the long *n*-decyl side chains was found to be slightly increased to 2.5 nm for the PNMG_124_-*b*-PNDG_63_ nanosheets as compared to that of the PNMG-*b*-PNDG nanofibrils and nanorods. Meanwhile, the (001) peak is slightly broader than those observed from PNMG_105_-*b*-PNDG_20_ nanofibrils and PNMG_121_-*b*-PNDG_46_ nanorods (2.4 nm), implying the more disordered molecular packing of PNDG backbones along the crystallographic c-axis in the PNMG_124_-*b*-PNDG_63_ nanosheets presumably due to the backbone folding.

The above results show that the solution self-assembly of coil-crystalline diblock copolypeptoids highly relies on the volume fraction of the crystallizable PNDG segment relative to that of the solvophilic PNMG segment. With increasing volume fraction of the PNDG block, the final aggregate morphology gradually transits from long wormlike nanofibrils, to short rigid nanorods and then to 2D nanosheets. Here, the self-assembly pathway of PNMG-*b*-PNDG plays a key role in determining the final aggregate morphology. SAXS (Figure 6a–c) and cryo-TEM analysis [79] revealed the formation of spherical micelles of PNMG_121_-*b*-PNDG_46_ in methanol at 65 °C with a *R*_g_ of 14.9 nm and an aggregation number of ~175. This is in sharp contrast to the PNMG_105_-*b*-PNDG_20_ methanol solution in which all polymers exist as unimers (*R*_g_ = 2.3 nm) at 65 °C. Note that no crystallization of PNDG block was observed at this temperature. We attributed the large difference in the initial association state to the solubility difference of these polymers in methanol: With longer PNDG segments, the stronger solvophobic interaction drives the micellation of diblock copolypeptoids at high temperature (i.e., T > T_m_), forming amorphous spherical micelles. Using SAXS/WAXS, we also found that subsequent cooling the solution to room temperature immediately induces the crystallization of PNDG segments within the micellar core. As dissociation of micelles upon cooling to room temperature is highly unlikely, the pre-formed spherical micelles of PNMG_121_-*b*-PNDG_46_ must undergo confined crystallization of PNDG within the micellar core upon cooling [15,16]. If we consider the length of the PNMG_121_-*b*-PNDG_46_ nanorods to be ~200 nm in average at the final state, as evidenced by cryo-TEM (Figure 6d), the aggregation number of the PNMG_121_-*b*-PNDG_46_ nanorods is then estimated to be ~1834. This number is about 10 times larger than that of the amorphous spherical micelle precursor (~175) at 65 °C prior to the onset of crystallization, which clearly indicates that the crystallization-induced fusion and structural rearrangement of preformed spherical micelles must have occurred to yield the final nanorods. Therefore, the self-assembly pathway for the PNMG_121_-*b*-PNDG_46_ nanorods, as depicted in Figure 6h, is distinctly different from the aforementioned self-seeding growth of PNMG_105_-*b*-PNDG_20_ nanofibrils (Figure 5j).

We shall also mention that the scenario becomes more complicated for the PNMG_124_-*b*-PNDG_63_ nanosheets and the detail formation mechanism of these nanosheets remains somewhat ambiguous. Based on SAXS/WAXS and cryo-TEM results, PNMG_124_-*b*-PNDG_63_ molecules aggregate into large non-spherical, amorphous clusters even at high temperature presumably due to the strong solvophobic effect. Upon cooling, PNDG segments are recrystallized within these large aggregates, which introduces an additional crystallization driving force for subsequent fusion/reorganization of preexisting aggregates. However, fusion/reorganization can be a rare event if the number of preexisting micelles per unit volume is too low. The existence of large aggregates due to strong solvophobic effect may thus poses an obstacle to the formation of well-defined polypeptoid nanostructures via CDSA. As will be described in the following section, the self-assembly pathway and final aggregate morphology of diblock copolypeptoids become more defined when the PNDG is replaced by PNOG, i.e., a crystallizable but less solvophobic block.

### 3.4. Effect of Side Chain Branching on the Solution Self-Assemblies

**Figure 7:**
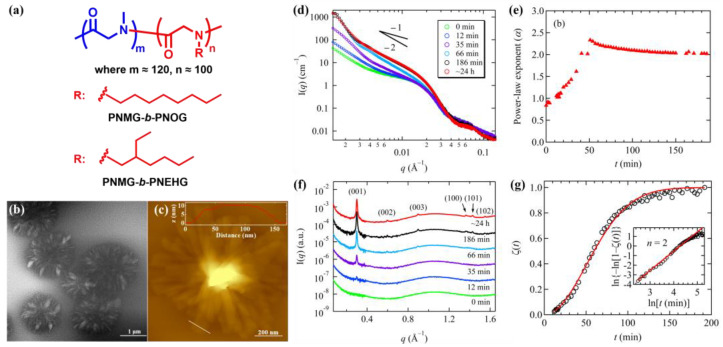
(**a**) The chemical structures of PNMG-*b*-PNOG and PNMG-*b*-PNEHG used for comparison purpose. (**b**) Cryo-TEM and (**c**) AFM images for PNMG_116_-*b*-PNOG_94_ in a diluted methanol solution. (**d**) Representative SAXS profiles of the 5 mg/mL PNMG_116_-*b*-PNOG_94_ methanol solution at different waiting times (*t*) after being cooled down to room temperature. (**e**) Plot of the exponent (*a*) values of *I*(*q*) ~ *q*^−*a*^ near *q* = 0.006 Å^−1^ as a function of *t*. (**f**) The corresponding WAXS profiles at different *t*, where the data have been shifted vertically for clarity. (**g**) The ζ(*t*) values (black circles) obtained from the normalized integrated intensity of the (001) peak at different *t*. Inset of (**g**) shows the corresponding Sharp–Hancock plot. The red solid lines in (**g**) correspond to the best-fits to the data using the Avrami–Erofeev expression, ζ(*t*) = 1–exp[–(*kt*)*^n^*], with *n* = 2. Figure reproduced from reference [84] with permission from the American Chemical Society.

As we mentioned in Section 2.2, asymmetric branching of the aliphatic N-substituents has profound impact on the molecular packing and phase behavior of polypeptoid homopolymers. To understand how side chain branching further influences the solution self-assembly of amphiphilic diblock copolypeptoids, two types of diblock copolypeptoids, i.e., PNMG_116_-*b*-PNOG_94_ and PNMG_121_-*b*-PNEHG_101_ (Figure 7a), with nearly identical molecular weight (i.e., *M*_n_ ≈ 25 kDa) and volume fraction of solvophobic block (*f_PNOG_* = 0.73, *f_PNEHG_* = 0.74) were recently investigated for their solution self-assembly behavior in methanol. Both samples were first heated at high temperatures, allowing polymers to be fully dissolved and existed as unimers in methanol. The solution self-assembly were then triggered by cooling of the respective methanol solution from high temperature to room temperature.

As seen in Figure 7b,c, PNMG_116_-*b*-PNOG_94_ molecules bearing linear *n*-octyl side chains self-assembled into large hierarchical microflowers that comprised of radially arranged nanoribbon subunits (i.e., flower petals) after ~24 h of assembly time. Such morphological feature at the final stage of self-assembly has also been captured by solution SAXS, which gives I(*q*) ~ *q*^−2^ power law behavior at the intermediate *q* range and followed by an intensity minimum at *q* ~ 0.045 Å^−1^ (Figure 7d). The highly ordered crystalline structure of PNOG blocks with a typical board-like molecular geometry was also revealed by WAXS, where the molecules are fully extended in an all cis-amide conformation and are stacked side by side and face to face simultaneously. Time-dependent SAXS/WAXS (Figure 7d–g) and AFM results [84] show that the overall self-assembly process is relatively sluggish and involves the assembly of multilevel building blocks in a stepwise fashion: Upon cooling, the PNMG_116_-*b*-PNOG_94_ unimers first associate to form amorphous spherical micelles owing to the high solvophobic content; These amorphous micelles are further aggregated via a nucleation-and-growth mechanism, resulting in the formation of flower petal junction; Finally, the growth of the flower petals (i.e., nanoribbon sub-units) occurs by the continuous addition of the amorphous PNMG_116_-*b*-PNOG_94_ materials to the crystallization front following a 2D crystallization kinetic, evidenced by the Avrami analysis (Figure 7g). For a 5 mg/mL solution, the entire self-assembly process takes few hundreds of minutes to complete. The relative sluggish self-assembly process (compared to PNMG_121_-*b*-PNEHG_101_ counterpart) is mainly attributed to the slow epitaxial 2D crystalline growth of board-like PNOG segments during micellar fusion/reorganization process, resulting in the formation of long-range ordered crystal lattice at molecular level.

**Figure 8:**
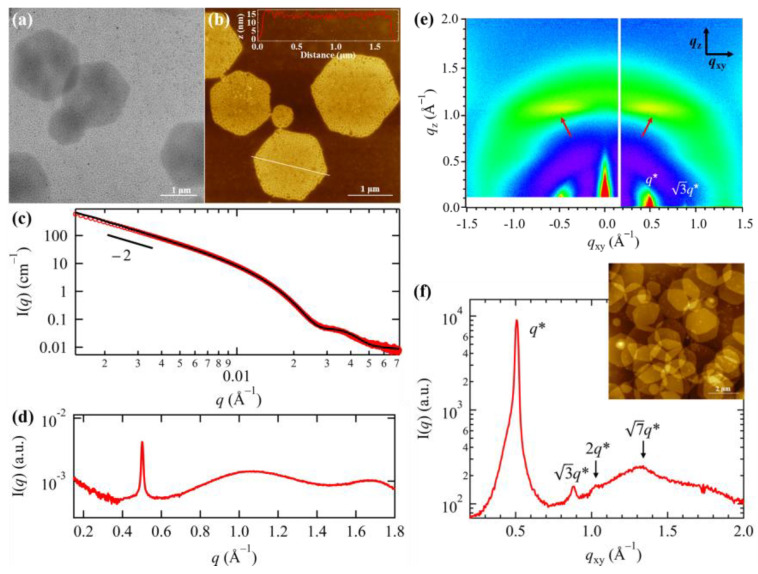
(**a**) Cryo-TEM and (**b**) AFM images for PNMG_121_-*b*-PNEHG_101_ in diluted methanol solution. (**c**) SAXS and (**d**) WAXS intensity profiles for the 5 mg/mL PNMG_121_-*b*-PNEHG_101_ in methanol. The solid line in (**c**) corresponds to the best-fit to the data based on the scattering form factor for disk-shaped polymer micelles. (**e**) Two-dimensional GIWAXD image for the PNMG_121_-*b*-PNEHG_101_ hexagonal nanosheets deposited onto a Si substrate. The two off-axis streaks are indicated by red arrows in (**e**). (**f**) One-dimensional GIWAXD profile along the *q*_xy_ (in-plane) direction. The corresponding AFM image for the GIWAXD sample is shown in the inset of (**f**). Figure reproduced from reference [84] with permission from the American Chemical Society.

By contrast, the PNMG_121_-*b*-PNEHG_101_ molecules bearing bulky branched racemic 2-ethyl-1-hexyl side chains were found to self-assemble into symmetric 2D hexagonal nanosheets (Figure 8a,b). The best-fit to the SAXS profile of the final PNMG_121_-*b*-PNEHG_101_ nanosheets using the scattering form factor for disk-shaped polymer micelles [123,124] gives a core thickness of 11.3 ± 0.2 nm and a radius of gyration of the corona chains of 2.7 ± 0.2 nm. The total thickness of the hexagonal nanosheets is then estimated to be 23.1 ± 1.0 nm, which is larger than that (16 ± 1 nm) estimated at the dry state by AFM analysis. Meanwhile, the WAXS profile of PNMG_121_-*b*-PNEHG_101_ nanosheets (Figure 8d) shows a single diffraction peak at q* = 0.50 Å^−1^, corresponding to the distance (1.26 nm) between adjacent PNEHG segments that are separated by the bulky branched N-2-ethyl-1-hexyl side chains [97]. Grazing-incidence wide-angle X-ray diffraction (GIWAXD) measurements were performed to unveil the molecular packing and orientation inside of the PNMG_121_-*b*-PNEHG_101_ nanosheets. Due to geometric confinement, the large 2D hexagonal nanosheets were laid flat on the Si substrate (inset of Figure 8f), allowing the molecular orientation within the dried hexagonal nanosheets to be resolved. Aside from the primary diffraction peak at q* = 0.50 Å^−1^, multiple higher order peaks located at √3q*, √4q*, √7q* along the in-plane (*q*_xy_) direction were also observed by GIWAXD (Figure 8e–f), indicating the rod-like PNEHG molecules are packed into a hexagonal lattice with the long axis of the rods aligned normal to the substrate (and the surface of hexagonal nanosheets). We speculate that the absence of these higher order peaks in Figure 8d is due to the relatively strong incoherent scattering from solution WAXS measurements using a capillary cell. There are also two discrete off-axis streaks with low intensity that are aligned parallel to *q*_xy_, as indicated by the red arrows in the GIWAXD (Figure 8e), possibly due to the presence of short and non-continuous helix-like segments along the backbone in low abundance.

**Figure 9:**
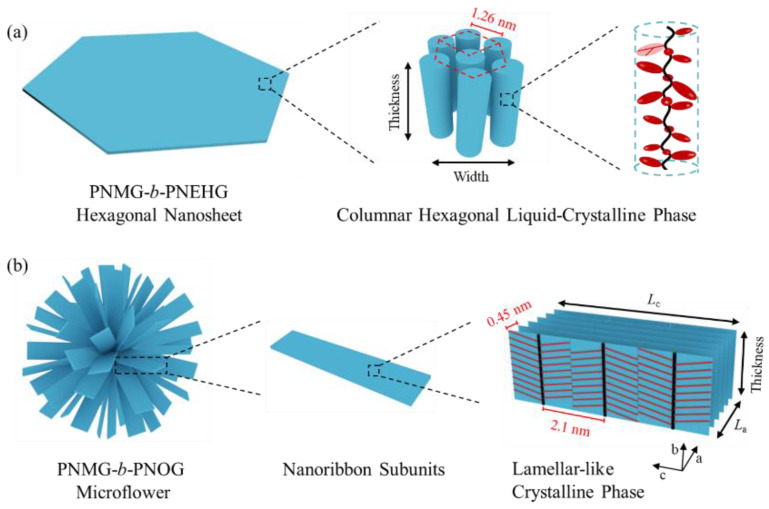
Schematic illustration of the molecular arrangement inside the (**a**) PNMG_121_-*b*-PNEHG_101_ hexagonal nanosheet and (**b**) PNMG_116_-*b*-PNOG_94_ microflower. The corona-forming PNMG blocks and possible chain folding of PNOG were omitted for clarity. Figure reproduced from reference [84] with permission from the American Chemical Society.

Hence, it is evident that the lateral dimension of the PNMG_121_-*b*-PNEHG_101_ nanosheet is governed by the preferential packing of rod-like PNEHG molecules in a columnar hexagonal lattice in 2D, whereas the thickness of the nanosheet core is determined by the height of hexagonal columns (Figure 9a). Within the columnar hexagonal mesophase, PNEHG blocks adopt a rod-like molecular geometry with an extended backbone conformation with the bulky racemic N-2-ethyl-1-hexyl side chains radially and outwardly displayed along the backbone. Interestingly, it was also found that the final PNMG_121_-*b*-PNEHG_101_ nanosheets were formed immediately (i.e., within seconds) after the solution was cooled below the clearing temperature, as evidenced by the little change of scattering profiles with time. Differs from the long-range ordered PNOG crystals, the intermolecular packing of the rod-like PNEHG blocks favors the formation of a columnar hexagonal LC mesophase which may not be very long ranged, evidenced by the weak higher order diffraction peaks even at the dried state (Figure 8f). This is consistent with DSC and WAXD results for the bulk PNEHG homopolymer with similar DP_n_ (Figure 4a,b). Thus, the formation of the mesophase within the PNEHG micellar core occurs much more rapidly relative to that of the crystalline PNOG micellar core presumably due to the less defined molecular packing structure in the former relative to the latter [37,128].

The correlations among aggregate morphology, molecular packing and N-substituent architecture (i.e., linear versus branched) of diblock copolypeptoid can be now rationalized. According to the general packing motif, a single PNOG board-like molecule in the crystalline lattice contains three different facets: The main face of PNOG that comprised of both backbone and N-aliphatic side chain, the surface comprised of PNOG backbone chain ends and the surface comprised of only N-aliphatic side chain ends, which are perpendicular to the crystallographic *a*-, *b*- and *c*-axes of the PNOG molecule, respectively (Figure 4c). Since PNOG blocks are covalently linked with the corona-forming PNMG blocks in the diblock copolypeptoids, it is reasonable that the PNOG crystals cannot grow along crystallographic *b*-axis. The Avrami analysis on the time-dependent WAXS results also showed that the crystalline growth of the core-forming PNOGs is two-dimensional (Figure 7g). We therefore postulate that the core thickness of the PNMG-*b*-PNOG nanoribbons is determined by the crystalline dimension along *b*-axis, while the other two axes determine the lateral dimension of the nanoribbon core (Figure 9b). Based on AFM analysis, the average length of the nanoribbons is approximately 4–5 times larger than their width, which suggests that the tendency for the core-forming PNOG block to grow along one axis is 4–5 times higher than the other axis. This is consistent with other previous studies [77,78,79,127] which show that the 2D nanosheets assembled from the diblock copolypeptoids bearing board-like crystallizable blocks from solution usually appears non-symmetrical, such as ribbon-like or rectangular shapes, rather than forming symmetrical 2D geometry. Yet, how these elongated 2D nanoribbons are stacked radially along flower petal junction and the detailed hierarchical self-assembly mechanism of the microflowers remain somewhat ambiguous. However, it is clear that the nanoribbon formation is due to the favorable molecular packing along one of the crystallographic axes during the 2D crystalline growth, which is dictated by the disparate inter- or intramolecular interactions and polymer–solvent interactions along different crystallographic axes.

By contrast, as the bulky racemic 2-ethyl-1-hexyl side chains are randomly distributed around the PNEHG backbone (Figure 4c), the rod-like PNEHG blocks would afford isotropic inter- or intramolecular interactions and polymer–solvent interfacial interaction in the radial direction of the rods. As the solvophilic PNMG block is chemically linked with PNEHG block, columnar hexagonal packing of PNEHG becomes energetically favored, resulting in the formation of large hexagonal nanosheets that possess a symmetrical 2D geometry (Figure 9a). We also found that the lateral dimension of PNMG_121_-*b*-PNEHG_101_ hexagonal nanosheets can be manipulated from nano-size (e.g., ~200 nm) to micro-size (e.g., ~2 μm) by tuning the initial polymer concentration within the dilute regime, while the thickness of hexagonal nanosheets remains unaffected by the concentration [84]. It should be noted that the symmetrical hexagonal nanosheets formed by PNMG_121_-*b*-PNEHG_101_ mesogens are highly unusual and rarely observed by crystallization or CDSA of typical crystalline polymers (e.g., polyethylene and polycaprolactone) [129,130,131]. This finding, which uses rod-like mesogens as the primary building block to induce the formation of 2D hexagonal nanosheets via solution self-assembly, sheds new light on the creation of highly symmetric 2D nano-/micro-scale materials for a wide range of applications.

## 4. Conclusions and Outlook

In this article, we review our recent experimental studies on the crystallization-driven solution self-assembly of amphiphilic diblock copolypeptoids bearing alkyl side chains. It has been found that supramolecular self-assembly and aggregate morphology of diblock copolypeptoids in solution are extremely sensitive to their molecular characteristics, such as block composition, molecular weight and N-substituent architecture. This is because micellation (solvophilic/solvophobic interaction), crystallization and the interplay between these two driving forces are directly linked to the detailed molecular characteristics of the diblock copolypeptoids. Because crystallization is more of a kinetically controlled process that can lead to non-equilibrium assembly, the spatiotemporal evolution of crystallizable BCPs during solution self-assembly must be carefully characterized in situ in order to gain a comprehensive understanding on the assembly pathway. Here, we highlight the use of in situ small-/wide-angle X-ray/neutron scattering in conjunction with other microscopic techniques in probing the molecular packing, hierarchical structure, and self-assembly pathways of crystallizable diblock copolypeptoids in solution, which allow us to better understand their multiscale structural evolution and self-assembly pathways. However, in priori design of solution self-assembly process to arrive at a targeted nanostructure/morphology still remain challenging.

Here, we listed several fundamental questions regarding diblock copolypeptoids bearing alkyl side chains that remain unsolved: (i) How do the interplay among inter-/intra-molecular interactions of board-like polypeptoids along different crystallographic axes dictate the anisotropic growth or the aspect ratio of 1D or 2D nanostructures. Can these interactions be mediated through chemical design or sample preparation protocols to achieve a tunable morphology? (ii) If chain folding is inevitable upon crystallization, in what direction (*a* or *c*-axis) do long polypeptoid segments prefer to fold inter-/intra-molecularly within the nanostructures? How this affects the chain conformation (or packing density) of solvophilic block, micellar fusion/reorganization, dispersibility and final aggregate morphology in solution? (iii) What lead to the radial stacking of nanoribbons to form hierarchical microflowers? How can we tune the structure and level of hierarchy based on the kinetic and thermodynamic behaviors of primary building blocks? How can we control each growth step that constitute the hierarchical assembly process? (iv) What is the self-assembly pathway and formation mechanism of hexagonal nanosheets composed of diblock copolypeptoids bearing asymmetrically branched alkyl side chains, e.g., PNMG-*b*-PNEHG? Can the use of mesogenic building blocks with less ordered LC-like packing serve as a new paradigm for the design of solution self-assemblies with unique structures and properties? Future efforts that incorporate predictive theoretical tools and advanced structural characterization methods would be helpful to address these issues.

Nevertheless, it is exciting to see that even for the simplest AB-type diblock copolypeptoids with alkyl side chains, a variety of well-defined non-spherical nanostructures with diverse morphology and hierarchy can be readily fabricated by CDSA, ranging from 1D nanofibrils, to nanorods, to 2D nanosheets and to hierarchical nanostructures. Owing to the recent advances in controlled synthetic methods, such as the submonomer solid-phase synthesis and sequential ROP of R-NCAs or R-NTAs, well-defined crystallizable block copolypeptoids with diverse N-substituent structure and tunable molecular sequences can now be produced with high efficiency, providing seemingly unlimited choices of polypeptoid building blocks for solution self-assembly. Besides, by thinking solution CDSA as a reaction process, recent findings on the living CDSA of several other BCPs (in particular polyferrocenylsilane-based polymers) that utilizes the “seeded-growth” protocol have envisioned a more precise control of size dispersity, complexity and hierarchy of polymeric self-assemblies [5,10,19,22,23,24,25,26,27,28,29]. Considering the unique biological properties of polypeptoid, these advancements would open new opportunities for the future design of novel polypeptoid nanomaterials with tailorable structure, property and functionality, which are potentially useful in molecular biomimicry and biomedical/biotechnological applications.

## Data Availability

The data are available from the corresponding author upon request.
